# Learning from human and chemical languages to predict biological function

**DOI:** 10.64898/2026.08.09.743788

**Published:** 2026-08-14

**Authors:** Clayton W. Kosonocky, Nikol Kadeřábková, Kangsan Kim, Ayesha J.S. Mahmood, Alexander Dunmyre, Phillip Woolley, Kristi Xing, Daniel Winkler, Tomer Babu, Filip Kadeřábek, Jonathan L. Sessler, Eric V. Anslyn, Edward M. Marcotte, Y. Jessie Zhang, Andrew D. Ellington, Despoina A.I. Mavridou

## Abstract

Understanding how molecular structure encodes biological function remains a grand challenge in drug discovery. Here, we present PubCheF-1, a deep learning model that predicts literature-derived biological function directly from chemical structure. PubCheF-1 was trained on a dataset linking molecules to labels derived from the scientific articles in which they appear, a strategy that connects disparate compounds through the language used to describe their functionalities. When tasked with identifying inhibitors of β-lactamases, including enzymes considered largely refractory to inhibition, PubCheF-1 predicted structurally distinct compounds that collectively have activity against all β-lactamase classes. Furthermore, hit compounds directly bind the enzyme active site, restore antibiotic efficacy in multidrug-resistant high-priority pathogens, and demonstrate potent activity in animal infection models. Together, these findings establish that machine learning-based prediction of biological function derived from the language of scientific literature allows the identification of bioactive molecules at high hit rates, thereby accelerating therapeutic discovery.

## Full Text Availability

The license terms selected by the author(s) for this preprint version do not permit archiving in PMC. The full text is available from the preprint server.

